# A Homolog of the Vaccinia Virus D13L Rifampicin Resistance Gene is in the Entomopoxvirus of the Parasitic wasp, *Diachasmimorpha longicaudata*

**DOI:** 10.1673/031.008.0801

**Published:** 2007-02-13

**Authors:** Pauline O. Lawrence, Barney E. Dillard

**Affiliations:** ^1^ Department of Entomology and Nematology, University of Florida, Gainesville, FL 32611; ^2^ Department of Surgery, University of Illinois at Chicago, Chicago, IL 60612

**Keywords:** DlEPV *rif gene*, wasp virus, symbiotic entomopoxvirus

## Abstract

The parasitic wasp, *Diachasmimorpha longicaudata* (Ashmead) (Hymenoptera: Braconidae), introduces an entomopoxvirus (DlEPV) into its Caribbean fruit fly host, *Anastrepha suspensa*. (Loew) (Diptera: Tephritidae), during oviposition. DlEPV has a 250–300 kb unipartite dsDNA genome, that replicates in the cytoplasm of the host's hemocytes, and inhibits the host's encapsulation response. The putative proteins encoded by several DlEPV genes are highly homologous with those of poxviruses, while others appear to be DlEPV specific. Here, a 2.34 kb sequence containing a 1.64 kb DlEPV open reading frame within a cloned 4.5 kb *Eco*R1 fragment (designated R1–1) is described from a DlEPV *Eco*RI genomic library. This open reading frame is a homolog of the vaccinia virus rifampicin resistance (*rif*) gene, D13L, and encodes a putative 546 amino acid protein. The DlEPV *rif* contains two *Eco*RV, two *Hind*III, one *Xba*I, and one *Dra*II restriction sites, and upstream of the open reading frame the fragment also contains *Eco*RV, *Hind*II, *Sp*EI, and *Bs*P106 sites. Early poxvirus transcription termination signals (TTTTTnT) occur 236 and 315 nucleotides upstream of the consensus poxvirus late translational start codon (TAAATG) and at 169 nucleotides downstream of the translational stop codon of the *rif* open reading frame. Southern blot hybridization of *Hind*III-, *Eco*RI-, and *Bam*H1-restricted DlEPV genomic DNA probed with the labeled 4.5 kb insert confirmed the fidelity of the DNA and the expected number of fragments appropriate to the restriction endonucleases used. Pairwise comparisons between DlEPV amino acids and those of the *Amsacta moorei*, *Heliothis armigera*, and *Melanoplus sanguinipes* entomopoxviruses, revealed 46, 46, and 45 % similarity (identity + substitutions), respectively. Similar values (41–45%) were observed in comparisons with the chordopoxviruses. The mid portion of the DlEPV sequence contained two regions of highest conserved residues similar to those reported for *H. armigera* entomopoxvirus rifampicin resistance protein. Phylogenetic analysis of the amino acid sequences suggested that DlEPV arose from the same ancestral node as other entomopoxviruses but belongs to a separate clade from those of the grasshopper- infecting *M. sanguinipes* entomopoxvirus and from the Lepidoptera-infecting (Genus B or Betaentomopoxvirus) *A. moorei* entomopoxvirus and *H. armigera* entomopoxvirus. Interestingly, the DlEPV putative protein had only 3–26.4 % similarity with RIF-like homologs/orthologs found in other large DNA non-poxviruses, demonstrating its closer relationship to the Poxviridae. DlEPV remains an unassigned member of the Entomopoxvirinae (http://www.ncbi.nlm.nih.gov/ICTVdb/Ictv/index.htm) until its relationship to other diptera-infecting (Gammaentomopoxvirus or Genus C) entomopoxviruses can be verified. The GenBank accession number for the nucleotide sequence data reported in this paper is EF541029.

## Introduction

The Entomopoxvirinae Subfamily (Family: Poxviridae) is comprised of three genera based on morphology, host range, and genome size of viruses infecting Coleoptera (Genus A or Alphaentomopoxvirus), Lepidoptera (Genus B or Betaentomopoxvirus), and Diptera (Genus C or Gammaentomopoxvirus). The Orthoptera-infecting *M. sanguinipes* entomopoxvirus is currently a temporary species within the Betaentomopoxvirus (ICTVdB 2004). Although entomopoxviruses have been isolated from the Hymenoptera, they have yet to be assigned a genus (King et al. 1998).

Evidence for a distant relationship between chordopoxviruses and entomopoxviruses was initially based on DNA sequence comparisons of genes encoding thymidine kinase (Gruidl et al. 1992), DNA polymerase (Mustafa and Yuen 1991), and nucleoside triphosphate phosphohydrolase I (Hall and Moyer 1991; Yuen et al. 1991). The rifampicin resistance gene (*rif*) [and the putative protein (RIF) it encodes] found in chordopoxviruses such as vaccinia (Niles et al. 1986), variola (Shchelkunov et al. 1993), and swinepox (Massung et al. 1993), also occurs in several entomopoxviruses (Winter et al. 1995; Osborne et al. 1996; Afonso et al. 1999; Bawden et al. 2000). The *rif* gene was considered to be highly conserved within, and characteristic of, the Poxviridae and thus, a unique monophylectic origin was suggested (Osborne et al. 1996). However, RIF-like sequences and certain other proteins assumed to be unique to poxviruses occur in some large double stranded eukaryotic DNA non-poxvirus families, suggesting that poxviruses and these double stranded DNA viruses share the same ancestry (Iyer et al. 2001), and probably that RIF is not characteristic of the Poxviridae alone.

In vaccinia, the RIF protein (D13L) (Moss 1996, 2001) localizes predominantly on the concave surface of the membrane cisternae of viral crescents and is presumed to be essential as a scaffold for the formation of the Golgi-derived membranes, characteristic of the early stages of virion assembly (Sodiek et al. 1994). Morphologically similar structures are highly conserved within the Poxviridae (Nile et al. 1986; Shchelkunov 1993; Massung et al. 1993; Winter et al. 1995; Moss 1996, 2001; King et al. 1998) and likely, serve a similar function.

We report here the sequencing and comparative analysis of a complete open reading frame within a partially sequenced clone (designated RI-1) derived from an *Eco*RI library of the *Diachasmimorpha longicaudata* entomopoxvirus (DlEPV) DNA. DlEPV was first described from the parasitic wasp *D. longicaudata* (= *Biosteres* = *Opius longicaudatus*) (Hymenoptera: Braconidae) and was shown to be transmitted to the larvae (hosts) of the Caribbean fruit fly, *Anastrepha suspensa* (Loew) (Diptera: Tephritidae) during oviposition by the wasp (Lawrence and Akin 1990). DlEPV invades the host's hemocytes where it replicates and exhibits the immature virus, intracellular mature virus, cell-associated virus, and extracellular enveloped virus forms (Lawrence 2002, 2005) known to occur in members of the Poxviridae (Moss 2001). DlEPV inhibits encapsulation by the host's hemocytes, thereby protecting the wasp's eggs and as such, is the first symbiotic entomopoxvirus described to date (Lawrence 2005). We show that the DlEPV D13L homolog is more closely related to entomopoxviruses and chordopoxviruses than to orthologs/paralogs of other large double stranded DNA viruses.

Few viruses or virus-like particles that are symbionts of parasitic wasps that attack dipteran hosts have been reported. The first virus-like particles from the *Leptopilina* parasitic wasp were reported from parasitized *Drosophila melanogaster* larvae and like DlEPV, were found to disrupt the cellular encapsulation ability of the host (Rizki and Rizki 1990). However, neither the nucleic acid composition nor family of these virus-like particles has been identified (Rizki and Rizki 1990). A rhabdovirus is also injected into *A. suspensa* larvae by the *D. longicaudata* female (Lawrence and Matos 2005) but its genes have also not been sequenced. Therefore, DlEPV is the first dipteran-infecting viral symbiont of a parasitic wasp for which any gene sequence is known.

## Materials and Methods

### Construction of the DlEPV EcoRI library

Details of the EcoRI DlEPV DNA library construction and sequencing of cloned fragments have been described (Lawrence 2002). Briefly, DlEPV DNA was extracted from virions that were harvested from female wasp venom glands and purified by sucrose density gradient centrifugation (Lawrence 2002). Upon digestion with EcoRI (Roche Molecular Biochemicals, www.roche.com), the resulting DlEPV DNA fragments were cloned into the pBluescript® II KS (+/-) cloning vector (pBS; Stratagene, www.stratagene.com ) using T4 DNA ligase (Roche) and the manufacturer's and standard (Sambrook et al. 1989) protocols. The clones were used to transfect supercompetent DH5-α *Escherichia coli* cells (Gibco-BRL, www.lifetech.com/www.invitrogen.com), amplified, and selected on ampicillin - Xgal (Gibco- BRL) agar plates at 37 °C for 18 h as previously described (Lawrence 2002). Recombinant plasmids were isolated from bacterial cells by alkaline lysis (Sambrook et al. 1989) and the presence of the DlEPV DNA inserts verified by EcoRI digestion and subsequent electrophoresis (Lawrence 2002). The clones (RI) were arbitrarily numbered and the RI-1 clone was selected for further analysis.

### DNA labeling, hybridization, and detection

To verify the fidelity of the RI-1 DNA insert to the DlEPV genome, a 3 µg sample of the isolated insert was labeled with digoxigenin (DIG) by random priming using the DIG-High Prime® labeling protocols (Roche). DlEPV genomic DNA was digested with *Eco*RI, *Hind*III, and *Bam*HI (Roche) and the resulting fragments electrophoresed into a 0.8% agarose gel at 30 V for 18 h and transferred to nitrocellulose membrane by the capillary method. The DNA was then fixed to the membrane by UV cross-linking at 50 mJoules. The blot was probed with 100 ng of the DIG-RI-1 insert diluted in 5 µl hybridization buffer [5X SSC (750 mM NaCl, 75 mM sodium citrate solution, pH 7.0), 0.1% (w/v) N-lauroylsarcosine, 0.2% (w/v) SDS, 1% blocking reagent (Roche)] at 65°C for 16 h. Hybridization was followed by two 5 min washes at RT with 2x washing buffer (2x SSC, 0.1% SDS) and two 15 min washes with 0.5x washing buffer. The hybridization signal was visualized using the DIG chemiluminescent detection protocol and exposure to LumiFilm (Roche).

### Sequencing of the open reading frame within the DlEPV RI-1 clone

Forward and reverse sequencing of the open reading frame within the RI-1 clone were accomplished by primer walking, with fluorescence-labeled dideoxynucleotides and *Taq* DyeDeoxy terminator cycle sequencing protocols (Applied Biosystems, Perkin-Elmer Corp., home.appliedbiosystems.com) and the extension products analyzed with a model 377A DNA sequencer (Applied Biosystems), as previously described (Lawrence 2002). Sequences were assembled and further analyzed with the Sequencher 3.0 software (Gene Codes Corp., www.genecodes.com).

### Sequence analysis of the RI-1 open reading frame

The amino acids deduced from the partial sequence of RI-1 by the Sequencher program were compared with homologs in the GenBank, PIR, and SWISS-PROT databases using the Basic Local Alignment Search Tool (BLAST) (Altschul et al. 1990). A multiple sequence alignment of the RI-1 open reading frame protein and its homologs was performed using the CLUSTALW 1.81 program (Thompson et al. 1994), with gap initiation and extension penalties of 10 and 0.2, respectively. Aligned sequences were imported into the Phylogenetic Analysis Using Parsimony (PAUP*®) program (Swofford 1998) to generate a phylogenetic tree using the neighbour joining method and 1,000 bootstrap trials to assess tree reliability. Pairwise comparisons of the DlEPV RI-1 open reading frame nucleotides and deduced amino acids with those of homologs identified by BLAST, were expressed as percent nucleotide identities, amino acid identities, or amino acid similarities [identities + homologous (conservative, *sensu*Mount 2001) substitutions].

**Figure 1.**
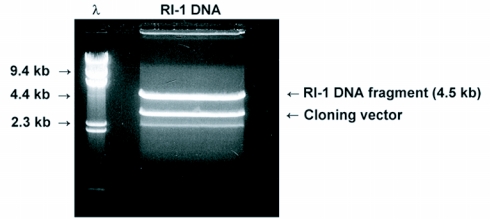
Electrophoretic analysis of the *Eco*RI digested DlEPV RI-1 clone. A 75 µl aliquot of the digested clone was applied to the gel. DNA fragment sizes were verified using a BioRad® λ high molecular weight DNA size standard (λ). The upper band corresponds to the RI-1 insert of approximate 4.5 kb. The lower band is the pBluescript® cloning vector of 2.96 kb.

Rifampicin-like proteins occur in other large DNA non-poxvirus families including the insect-infecting Iridoviridae and Ascoviridae (Iyer et al. 2001; Stasiak et al. 2001 Stasiak et al. 2003). Thus pairwise amino acid comparisons, separate from those made with the poxviruses, were performed between the RIF sequence of DlEPV, orthologs/homologs from the insect iridovirus IIV-6, the *Diadromus pulchellus* ascovirus 4a (DpAV4a) from a parasitic wasp of the same name, and other non-pox DNA viruses.

## Results

### Purification, sequencing and analysis of the RI-1 insert

**Figure 2.**
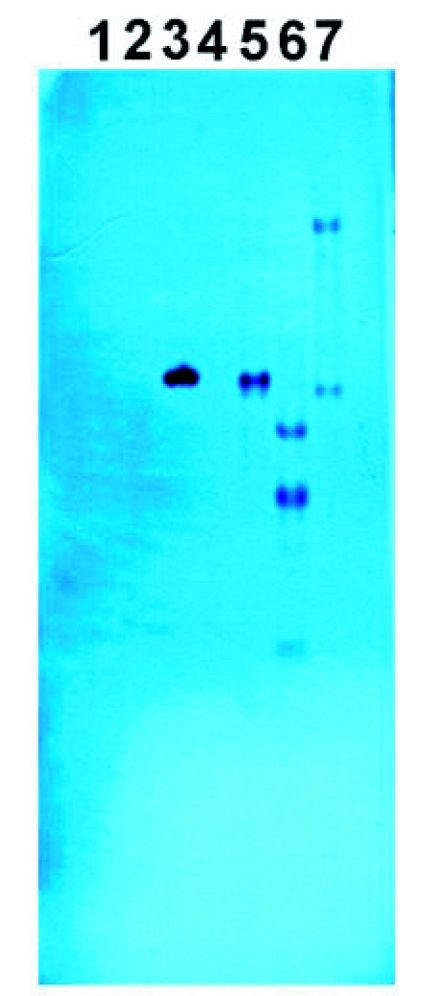
Autoradiograph of Southern hybridization of digested DlEPV genomic DNA with a 4.5 kb specific probe generated from the DlEPV R1-1 insert. Lanes 1–2: empty; Lane 3: 1 µl of the DlEPV R1-1 undigested 4.5 kb insert (positive control); Lane 4: 2 µl salmon sperm DNA (negative control); Lane 5: 5 µl *Eco*RI digested DlEPV genomic DNA; Lane 6: 5 µl *Hind*III digested DlEPV genomic DNA; Lane 7: 5 µl *Bam*HI digested DlEPV genomic DNA.

The size of the RI-1 insert was verified to be ∼ 4.5 kb (Figure 1). Hybridization of the DIG-probe to the insert and the restricted DlEPV genomic DNA in the Southern blot, verified their fidelity to the DlEPV genome (Figure 2). The single hybridized fragment, with the same size as the positive control (∼4.0), obtained with the *Eco*R1 digested genomic DNA confirmed the absence of an *Eco*R1 restriction site within the fragment (Figure 2). The four bands detected in blots of the *Hind*III digest (Figure 2) were also consistent with the presence of three *Hind*III sites within the sequence (Figure 3). Although no *Bam*HI sites (therefore one band) were predicted, two bands were observed (Figure 2), suggesting the presence of a second site in the unsequenced portion of the clone. Sequencher also predicted *Xba*I, *Dra*II, *Spe*I, and *Bsp*106 restriction sites within the RI-1 fragment (Figure 3) but these enzymes were not evaluated.

The sequenced portion of the RI-1 fragment was determined by Sequencher to contain one complete open reading frame of 1,640 bases, encoding a putative protein of 546 amino acids and an apparent partial open reading frame. The *rif* open reading frame had 529 bases (5′) and 174 bases (3′) immediately flanking its translational start and stop codons, respectively (Figure 3). Thus, the sequenced portion of R1-1 comprised 2.34 kb (GeneBank accession # EF541029) of the ∼4.5 kb R1-1 insert. The analyses below will focus only on the complete open reading frame and sequences immediately flanking it (Figure 3).

The translation initiation codon (ATG) of the open reading frame starts at 530 nucleotides from the 5′ end of the fragment and the translational stop codon (TAA) starts at 2,168 nucleotides (Figure 3). Immediately preceding the translational initiation codon is a highly A/T rich (87%) 30 nucleotide sequence. Three of these bases immediately preceding the ATG and in combination with it, form the consensus poxvirus late transcriptional start signal (TAAATG) (Rosel et al. 1986; Moss 1996, 2001) (Figure 3). Potential poxvirus early transcription termination signals (TTTTTnT) occur at 236 and 315 nucleotides upstream of the late translational start codon and 168 nucleotides downstream of the translational stop codon of the open reading frame (Figure 3).

Alignment of all deduced poxvirus sequences revealed almost no conserved amino acids within the first 253 amino acids of the DlEPV sequence, except for a short region [LPE(I)/(V)KG] between amino acids 53–58 in which valine was substituted in the chordopoxviruses for isoleucine in the entomopoxviruses (Figure 4a). Two additional motifs, HTN(L)/(I)/(V)L(M)/(V)/(S)F(GT)/(SR)/(TR)R and GD(N)/(L)RS, occur within DlEPV amino acids 326–370 (region I) and 383–441 (region II) respectively (Figure 4a). These regions of 43 and 58 amino acids have ∼28 and 26% conserved residues respectively, and correspond to the same two regions in the *H. armigera* entomopoxvirus RIF that had 56 and 53% conserved amino acids respectively, when that virus was aligned with vaccinia and swinepox (Osborne et al. 1996). When only entomopoxviruses were aligned, the conserved amino acids in regions I and II of the DlEPV RIF increased to ∼44 and 38% respectively (Figure 4b). Interestingly, when each entomopoxvirus sequence was individually aligned with DlEPV, the percent conserved residues increased even further to as high as 79 and 41% in regions I and II respectively (alignment not shown). In addition at least 10% of 40 residues at the N-terminus and 20% of 50 residues toward the C-terminus were conserved between DlEPV and each of the other (beta) entomopoxviruses (data not shown).

**Table 1.**
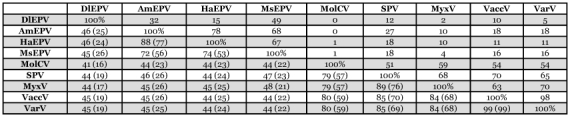
Pairwise comparison of amino acids and nucleotides of the rifampicin resistance homologs of DlEPV and other poxviruses. The lower left triangle represents the percent similarities (= amino acid identities plus homologous substitutions). Numbers in parentheses represent percent amino acid identities. The upper right triangle represents percent nucleotide identities.

**Figure 3a.**
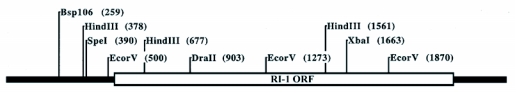
Locations of restriction enzyme recognition sites within a ∼2.54 kb sequenced portion of the RI-1 DNA fragment predicted by the Sequencher 3.0 program.

Regions I and II had motifs common to both chordopoxviruses and entomopoxviruses but contained substitutions that distinguished the two virus subfamilies (Figure 4a). A closer analysis of the entomopoxviruses revealed that within the motif in region I, DlEPV had a single substitution that distinguished it from the betaentomopoxviruses (Figure 4b). However, all residues in the motif in region II were conserved among all entomopoxviruses (Figure 4b).

**Figure 3b.**
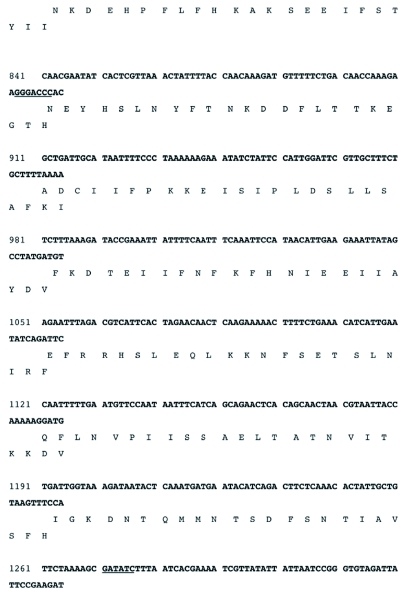
DNA sequence of the RI-1 open reading frame and an immediately preceding region (539 nt) containing putative poxvirus early transcriptional stop (TTTTTnT) and late promoter (TAAATG) sequences (highlighted in black). Restriction enzyme recognition sites, shown in (a), are underlined. The putative translational stop codon (TAA) is indicated by an asterisk (*). The sequence has been assigned GeneBank accession # EF541029.

**con't:**
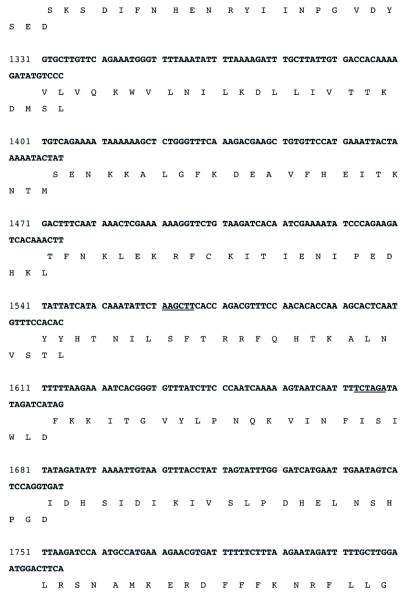

**con't:**
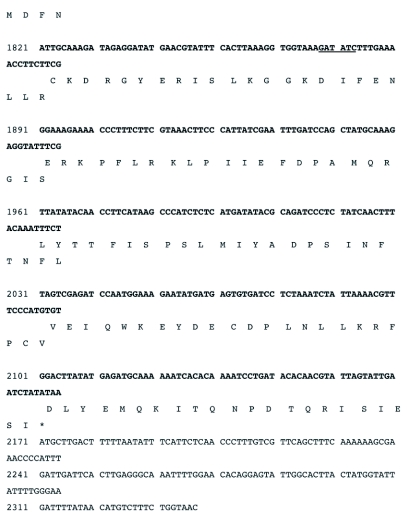

**Figure 4a.**
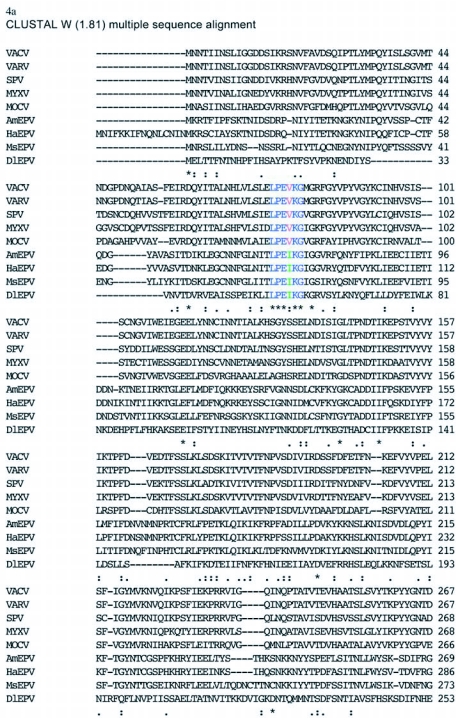
ClustalW 1.81 multiple sequence alignment of the deduced amino acid sequence of the putative rifampicin resistance protein homologs from *Amsacta moorei* entomopoxvirus (AmEPV), *Heliothis armigera* entomopoxvirus (HaEPV), *Melanoplus sanguinipes* entomopoxvirus (MsEPV), *Molluscum contiguosum* poxvirus (MOLCV), swinepox virus (SPV), Myxoma poxvirus (MYXV), vaccinia virus (VACV), variola virus (VARV), and *Diachasmimorpha longicaudata* entomopoxvirus (DlEPV). A colon (:) represents amino acid homologous (“conservative”, sensu Mount 2001) substitutions. A period (.) identifies amino acid non-homologous substitutions. Asterisks indicate identical amino acids conserved in all sequences. Underlined sequences represent regions I and II in HaEPV and DlEPV with the highest percent conserved amino acids previously identified for HaEPV by Osborne et al. (1996). For the three motifs identified within the RIF sequence, Blue = conserved in all poxviruses; Red = conserved only among chordopoxviruses; Green = conserved only among EPVs. Other colors = conserved in some members of a subfamily.

**con't:**
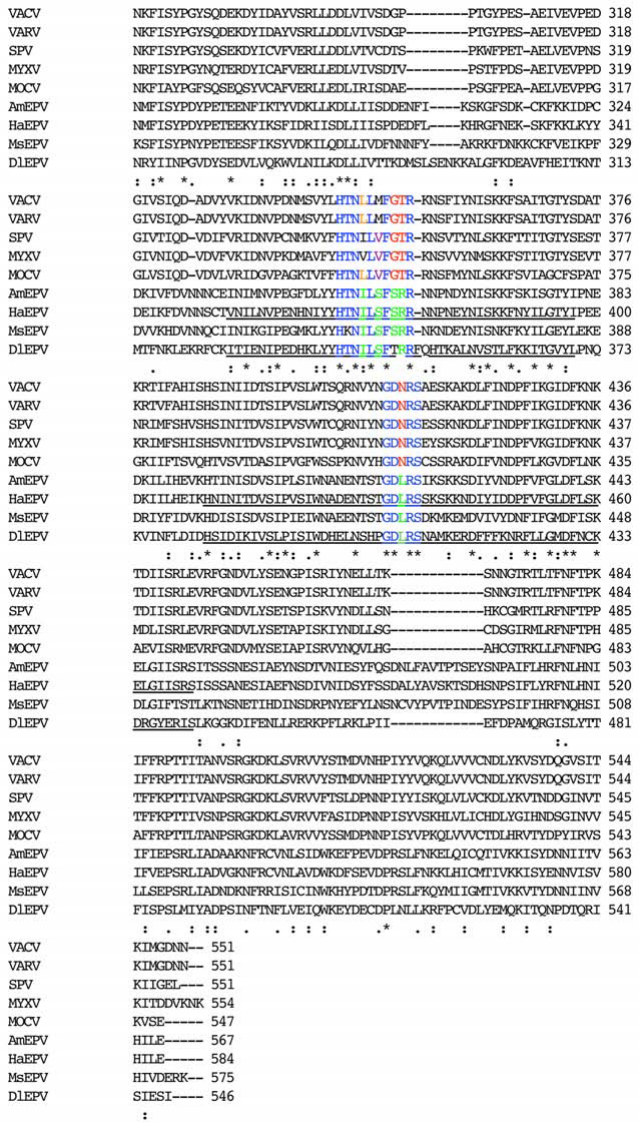

**Figure 4b.**
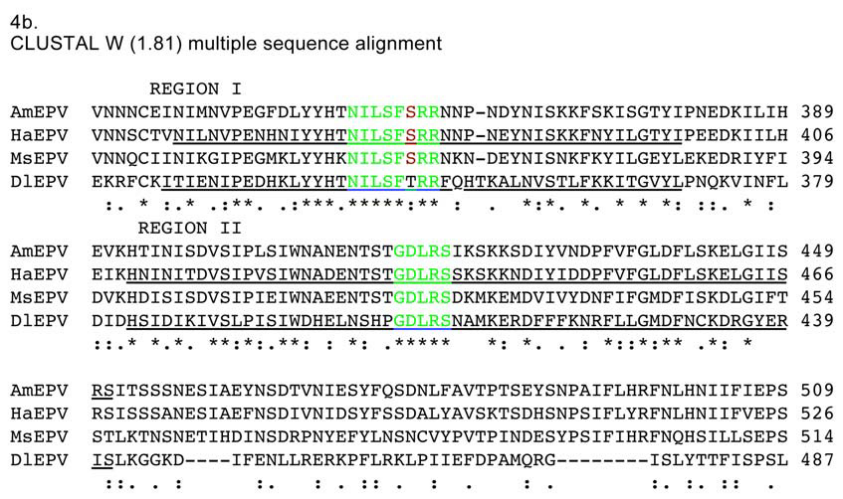
ClustalW 1.81 multiple sequence alignment of the deduced amino acid sequence of a selected region of the putative rifampicin resistance protein homologues from entomopoxviruses, showing regions I and II (underlined in HaEPV and DlEPV) of highest percent conserved sequences (Osborne et al. 1996) and their component motifs. Virus names, symbols, and color codes are as described in Fig. 4a.

Overall, pairwise comparison of amino acids of DlEPV RIF with each homolog revealed that DlEPV shared slightly more amino acid identities with the betaentomopoxviruses than with chordopoxviruses (Table 1). However, the betaentomopoxviruses shared 1.5–2 times more amino acids among themselves than they did with DlEPV and the lepidopteran entomopoxviruses shared more with each other than they did with the *M. sanguinipes* entomopoxvirus (Table 1). The percent similarities between DlEPV and all poxvirus RIF sequences and between the betaentomopoxviruses and chordopoxviruses were about the same (on average ∼44%) (Table 1). However, similarities among the betaentomopoxviruses were 1.5– 2 times higher than with DlEPV. The lepidopteran entomopoxviruses had greater similarity with each other than with the *M. sanguinipes* entomopoxvirus (Table 1).

The nucleotides conserved between DlEPV and the betaentomopoxviruses were 1.5 to > 5X fewer than those conserved among the betaentomopoxviruses themselves, with the lepidopteran entomopoxviruses sharing more with each other than with the *M. sanguinipes* entomopoxvirus (Table 1). Nevertheless, both DlEPV and the betaentomopoxviruses had few (0-≤ 20%) nucleotide identities with the chordopoxviruses, except in the case of the *A. moorei* entomopoxvirus and swinepox (Table 1). Thus, the DlEPV putative RIF protein is closer to (but distinct from) homologs of the lepidopteran and orthopteran entomopoxviruses than to those of chordopoxviruses (Table 1). This is further seen in the phylogenetic tree that assigns DlEPV to a different clade from the *M. sanguinipes* entomopoxvirus and from the *H. armigera* and *A. moorei* entomopoxviruses (Figure 5). DlEPV had ∼20% and 26.4% similarity respectively, with IIV-6 and DpAV4a, two non-pox double stranded DNA viruses of insects ≤22.96 with non-pox double stranded DNA viruses of other organisms (Table 2).

## Discussion

An EcoRI (RI-1) clone selected from a DNA genomic library of DlEPV from the parasitic wasp *D. longicaudata*, contains a complete open reading frame that was shown by BLAST search to be a homolog of the vaccinia *rif* (D13L) gene. Upstream of the *rif* open reading frame were characteristic poxvirus early transcription termination signals (TTTTTnT) (Moss 1996, 2001) (Figure 3). The presence of the characteristic poxvirus consensus late transcriptional start signal (TAAATG) and stop codons confirm that the DlEPV open reading frame is a late gene (Rosel et al. 1986). An 87% A/T rich region immediately before the DlEPV *rif* putative translational initiation site (Figure 3) is similar to the 91% adenylated sequence upstream of the translational start site in the *rif* of the *H*.*armigera* entomopoxvirus (Osborne et al. 1996).

The DlEPV RI-1 open reading frame is 1,641 base pairs and potentially encodes a 546 amino acid polypeptide that shares considerable similarity with RIFs of both chordopoxviruses and entomopoxviruses (Figure 4, Table 1). In vaccinia, RIF has been shown to be involved in the formation of the Golgi-derived crescent-shaped membranes characteristic of the early stages of virion assembly (Sodiek et al. 1994). Similar crescents also occur during DlEPV morphogenesis (Lawrence and Akin 1990). Because morphologically similar structures are conserved within the poxvirus family (Moss 1996, 2001) and are presumed to arise through similar mechanisms, RIF was considered to be unique to poxviruses (Osborne et al. 1996). However, there are reports of *rif*—like genes in certain other large DNA non-poxvirus families with which poxviruses are suspected to share a common ancestry (Iyer et al. 2001) but it is not clear whether they are functionally similar (Table 2). Amino acid comparisons between DlEPV and the insect-infecting non-pox DNA (asco- and irido-) viruses revealed ≤ 26.4% amino acid similarity among their RIF-like proteins, far less than the similarities between DlEPV and other poxviruses (Table 1). Thus while DlEPV RIF, like those of other poxviruses, may be distantly related to RIF-like proteins from non-pox large DNA viruses, it is closer to homologs of entomopoxviruses and chordopoxviruses (Table 2). These results, along with previously published phylogenetic comparisons of other DlEPV genes with those of other poxviruses (Lawrence 2002; Mwaengo and Lawrence 2003; Hashimoto and Lawrence 2005), further support our hypothesis that DlEPV is an entomopoxvirus.

**Table 2.**
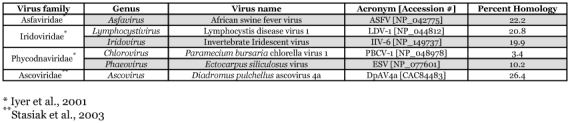
Percent similarity between DlEPV D13L vaccinia homolog and orthologs/homologs from large enveloped double stranded DNA viruses from non-poxvirus families.

The sequence alignment shows two highly conserved internal regions within DlEPV RIF that correspond to those described for the *H. armigera* entomopoxvirus (Osborne et al. 1996). Within these regions, two apparent motifs were evident but exhibited amino acid substitutions that were unique to their respective virus subfamilies (Figure 4a). Conserved inner regions of poxvirus RIFs have been hypothesized to interact with eukaryotic subcellular elements (Osborne et al. 1996). It has been further hypothesized that protein function may depend on their ‘head to tail’ interaction (Baldick and Moss 1985). The DlEPV deduced protein sequence showed very low amino acid conservation within its terminal regions in alignments with all poxviruses (Figure 4a) but had at least 10 and 20% conserved amino acids within 40 and 50 residues respectively, of the N-and C- termini in alignments with individual entomopoxviruses (data not shown). It is not clear whether or how these conserved amino acids at the DlEPV RIF termini may influence protein function within the host.

The present study demonstrates that DlEPV, a unique viral symbiont of a parasitic wasp of tephritid fruit flies, possesses yet another homolog of a poxvirus gene. While several DlEPV genes remain to be sequenced and characterized, almost 50% of sequences published to date (Lawrence 2002; Mwaengo and Lawrence 2003; Hashimoto and Lawrence 2005), collectively have the highest homology with those of entomopoxviruses. However, these DlEPV genes and deduced proteins exhibit sufficient differences from the lepidopteran and *M. sanguinipes* entomopoxviruses, that they were placed in a different entomopoxvirus clade (Figure 5), suggesting that DlEPV belongs to a different genus. DlEPV is designated as an unassigned species within the subfamily [00.058.2.00.001.00.001. *Diachasmimorpha entomopoxvirus* (DIEV) (ICTVdB 2004)] but its pathogenicity to dipterans (Shi et al. 1999; Lawrence 2005) suggests that it is likely a member of the Gammaentomopoxvirus genus. Its true phylogenetic position within the subfamily is hampered by the lack of sequences from known dipteran entomopoxviruses and therefore awaits further clarification.

## **Abbreviations**

DlEPV: Diachasmimorpha longicaudata entomopoxvirus;
Rif: rifampicin resistance gene;
RIF: putative rifampicin resistance protein
