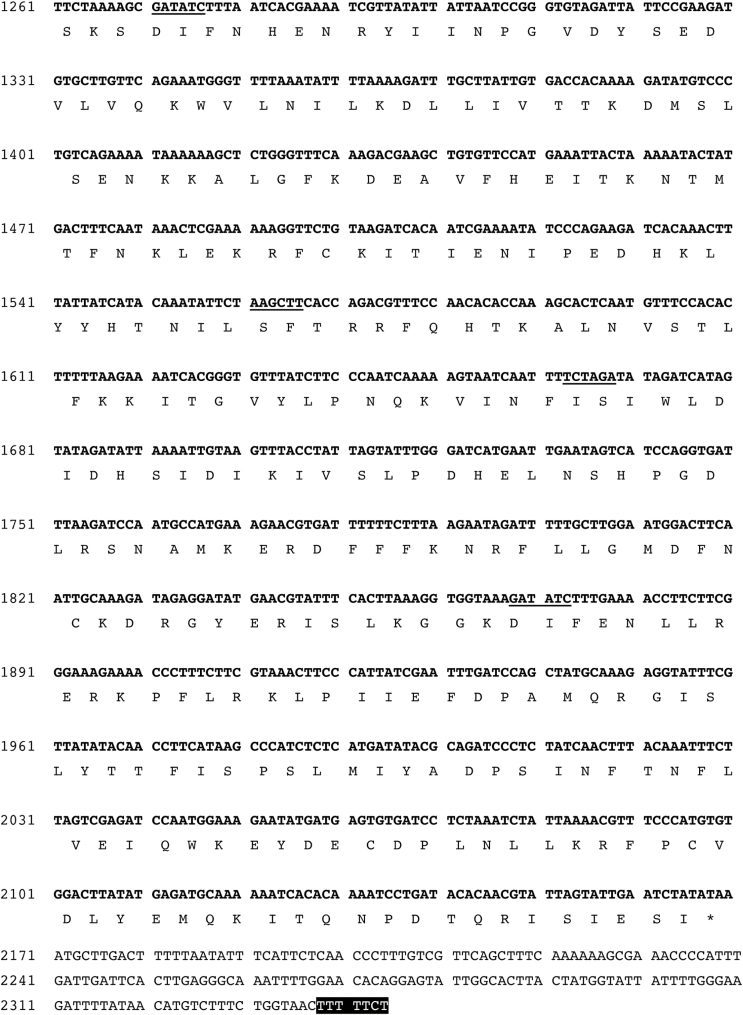# Erratum to: “A homolog of the vaccinia virus D13L rifampicin resistance gene is in the entomopoxvirus of the parasitic wasp”

**DOI:** 10.1093/jisesa/iex085

**Published:** 2017-12-09

Correction of “Lawrence, P. O., B. E. Dillard, III. 2007. A homolog of the vaccinia virus D13L rifampicin resistance gene is in the entomopoxvirus of the parasitic wasp, *Diachasmimorpha longicaudata*. J. Insect. Sci. 8(1): 8.”

DOI: 10.1673/031.008.0801

In this article, Figure 3b was truncated in the first published version. The corrected figure is below. The original article PDF was republished by the journal’s previous publisher in 2008 to include the corrected figure, but the full-text article was not updated at that time. Figure 3b has now been corrected in the full-text article, so the PDF and full-text article versions match.

**Fig. 3B.**
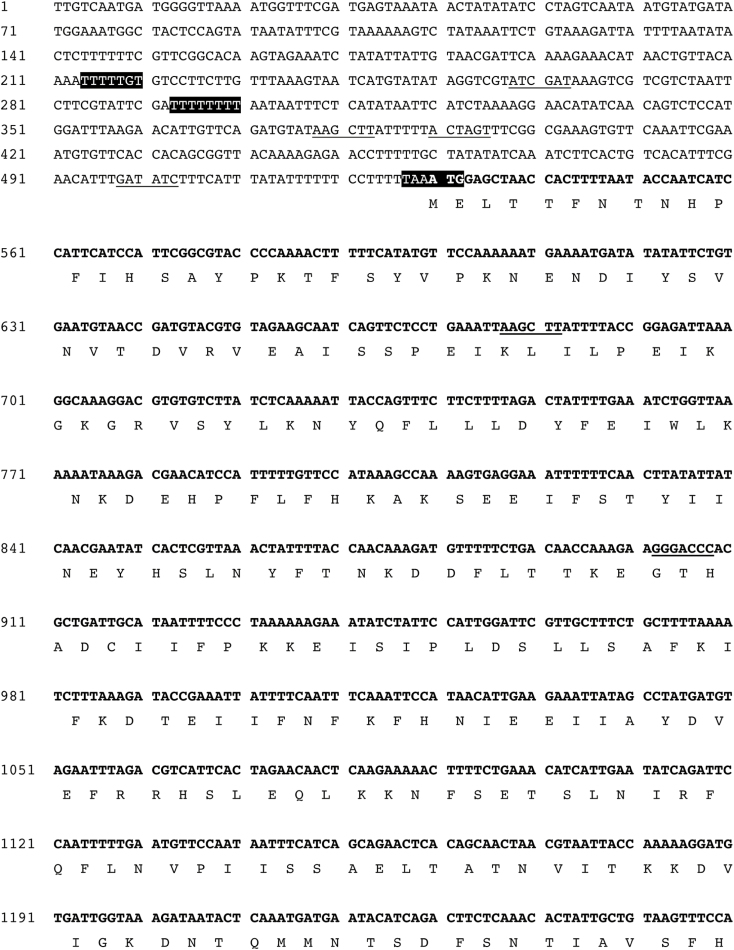


**Fig. 3B.**