# Supplementary material for: A Homolog of the Vaccinia Virus D13L Rifampicin Resistance Gene is in the Entomopoxvirus of the Parasitic wasp, Diachasmimorpha longicaudata
Source: J Insect Sci. 2007 Feb 13;8:8. doi: 10.1673/031.008.0801 (PMC3061580; doi:10.1673/031.008.0801)
Supplement: Related texts

# Correction

Figure 3b was originally published in a truncated form; the corrected version is shown below.

**Figure 3b.** DNA sequence of the RI-1 open reading frame and an immediately preceding region (539 nt) containing putative poxvirus early transcriptional stop (TTTTTnT) and late promoter (TAAATG) sequences (highlighted in black). Restriction enzyme recognition sites, shown in (a), are underlined. The putative translational stop codon (TAA) is indicated by an asterisk (*). The sequence has been assigned GeneBank accession # EF541029.

**continued:**
